# Editorial for the Special Issue on Advances in Micro and Nano Manufacturing: Process Modeling and Applications, Volume II

**DOI:** 10.3390/mi15060687

**Published:** 2024-05-24

**Authors:** Davide Masato

**Affiliations:** Department of Plastics Engineering, University of Massachusetts Lowell, Lowell, MA 01854, USA; davide_masato@uml.edu

In the second volume of the Special Issue on ‘Advances in Micro and Nano Manufacturing: Process Modeling and Applications’, we continue to witness the dynamic evolution of micro- and nanomanufacturing technologies. These advancements are pivotal in facilitating product miniaturization and integrating novel functionalities [1]. Notably, the spectrum of materials under study has expanded to include a focus on composite materials, broadening the scope of research in the field. Additionally, there has been a noticeable increase in geographical participation (China (5), Germany (1), Belgium (1), South Korea (1), Italy (2), and the United States (1)), signifying the global impact and interest in this area of study [2]. Furthermore, modeling approaches have evolved to incorporate cutting-edge techniques such as machine learning, enhancing the ability to simulate intricate interactions between materials, manufacturing processes, and product properties [3]. As we delve deeper into the micro/nano scales, it becomes increasingly apparent that traditional manufacturing paradigms must adapt to address the unique mechanical, thermal, and fluid dynamics phenomena encountered in this domain. Despite these remarkable advancements, several challenges persist [4]. Key among these are the elucidation of material processing behaviors on reduced scales, the establishment of robust design criteria, the validation of process control and monitoring strategies, and the definition of rigorous quality control procedures [5,6]. As we embark on this journey of exploration and innovation, it is imperative that we collectively address these challenges to realize the transformative potential of micro- and nanomanufacturing technologies.

This Special Issue showcases ten state-of-the-art examples of modeling and simulation in micro- and nanomanufacturing processes. The papers published in this Special Issue explore the following micro- and nanomanufacturing processes: (1) laser texturing, (2) micro- and precision injection molding, (3) micro-milling, (4) grinding, (5) electro-discharge machining, and (6) additive manufacturing. The remaining paper focused on piezoelectric actuators for micro-manufacturing actuation [Contribution 1].

1. Laser Texturing: Femtosecond laser ablation of metals is a precise method used to create microfeatures on a material’s surface with a minimized heat-affected zone [7]. Vanwersch et al. developed and validated a pulse-based two-temperature model to predict ablated geometry based on material and laser parameters [Contribution 2]. The model was implemented for grooves with varying hatch pitches, numbers of passes, and scanning directions. The model was corrected using experimental data and the profile shape, depth, and width were accurately predicted. Gao et al. used a femtosecond laser to texture metallic molds for micro-injection molding [Contribution 3]. Different hierarchical texture geometries were obtained using direct laser ablation to generate micro-scale features; then, the ultrashort pulsed laser was used to create laser-induced periodic surface structures (LIPSSs). The hierarchical texture designs were selected to achieve wetting functionalization while keeping a low aspect ratio for easier replication during molding [8]. Indeed, the aspect ratio is the main factor limiting the replication of textured mold surfaces [9].
2. Micro- and Precision Injection Molding: Jung and Kim simulated the deformation of a hot runner manifold and nozzle assembly during operation to address potential leaks and premature failure for high-precision applications [Contribution 4]. The simulation results accurately predicted the gap between the manifold and the nozzle bushing. It was reported that the deformation as a result of the melt pressure did not exceed 12% of that achieved through thermal loading. The results are essential as hot runners play a significant role in precision molding applications [10] and sustainable plastics [11]. Gao et al. studied the replication of a textured surface with micro-molding of different polymers [Contribution 3]. The replication rate provided key information about the ability of the polymer to flow into the micro-scale cavities, creating the desired microfeatures on the plastic molded parts. The replication depended on the orientation of inserts for those with directional geometry. Higher hesitation and lower replication characterized the texture geometries that promoted air entrapment [12,13].
3. Micro-Milling: Liu et al. established a mathematical model to predict chatter vibration and improve the accuracy of micro-milling [Contribution 5]. The model considered the centrifugal force induced by the rotational speed, the gyroscopic effect, and the tool runout, which are the main parameters affecting the process [14]. Comparison with the experiments demonstrated good accuracy and allowed the investigation of model performance under different milling conditions. Zhou et al. developed a 2D mesoscopic-based model to predict the effect of particle shape when cutting SiC P/Al composites [Contribution 6]. Experiments were conducted to evaluate the effect of different particle geometries on the modeled material removal prediction. The results highlight the deformation of the particles during machining and their impact on the cutting force.
4. Grinding: Wang et al. conducted dry grinding experiments using an Fe-Cr-Co permanent magnet alloy to study the effect of processing conditions on surface roughness [Contribution 7]. The grinding force signals were correlated with the rotational speed of the grinding wheel and the surface roughness. The results indicated that increasing the grinding wheel speed could reduce the difference in grinding force between the peak and valley.
5. Electro-Discharge Machining: Quarto et al. proposed the use of an Artificial Neural Network (ANN), together with Particle Swarm Optimization (PSO) and a Finite Element Model (FEM), to predict the process performances for the Micro-Electrical Discharge Machining (micro-EDM) drilling process [Contribution 8]. A comparison of the different models suggested that the integrated ANN-PSO methodology is more accurate in performance prediction. However, a large amount of historical data was required for the ANN training. The FEM model was also more complex to set up due to the need for accurate material characterization and the high computational time [15].
6. Additive Manufacturing: Chen et al. studied ejection cycle time and droplet diameter prediction for E-jet printing [Contribution 9]. Different machine learning models were evaluated and compared using experiments to benchmark the performance for varying processing conditions. Mader et al. used Fused Deposition Modeling (FMD) 3D printing to manufacture PS microfluidic channels with dimensions as small as 300 µm and high transparency [Contribution 10]. Moreover, other functional chip designs were demonstrated. Cell culture experiments demonstrated cell adhesion and proliferation.

The Guest Editor extends their gratitude to all of the authors for their invaluable contributions to this Special Issue, advancing the diffusion and development of micro- and nanomanufacturing technologies. A thank you also goes out to the dedicated reviewers for their time and effort in enhancing the quality of the submissions. We cordially invite all researchers and practitioners to consider contributing to the forthcoming third volume of this Special Issue [16], where your insights will continue to enrich the discourse and advancements in this field.

## References

[1] Peercy P.S. (2000). The drive to miniaturization. Nature.

[2] Masato D., Lucchetta G. (2022). Advances in Micro and Nano Manufacturing: Process Modeling and Applications.

[3] Wuest T., Weimer D., Irgens C., Thoben K.-D. (2016). Machine learning in manufacturing: Advantages, challenges, and applications. Prod. Manuf. Res..

[4] Razali A.R., Qin Y. (2013). A Review on Micro-manufacturing, Micro-forming and their Key Issues. Procedia Eng..

[5] Fu M., Wang J. (2021). Size effects in multi-scale materials processing and manufacturing. Int. J. Mach. Tools Manuf..

[6] Qin Y. (2018). Integrated, multidisciplinary approaches for micro-manufacturing research, and new opportunities and challenges to micro-manufacturing. Proc. Inst. Mech. Eng. Part N J. Nanomater. Nanoeng. Nanosyst..

[7] Masato D., Piccolo L., Lucchetta G., Sorgato M. (2022). Texturing Technologies for Plastics Injection Molding: A Review. Micromachines.

[8] Krantz J., Caiado A., Piccolo L., Gao P., Sorgato M., Lucchetta G., Masato D. (2022). Dynamic wetting characteristics of submicron-structured injection molded parts. Polym. Eng. Sci..

[9] Hansen H., Hocken R., Tosello G. (2011). Replication of micro and nano surface geometries. CIRP Ann..

[10] Chen T.-C., Huang C.-T., Chiu Y.-C., Wang W.-D., Hsu C.-L., Lin C.-Y., Kao L.-W. (2013). Material saving and product quality improvement with the visualization of hot runner design in injection molding. Int. J. Precis. Eng. Manuf..

[11] Kazmer D.O., Masato D., Piccolo L., Puleo K., Krantz J., Venoor V., Colon A., Limkaichong J., Dewar N., Babin D. (2021). Multivariate modeling of mechanical properties for hot runner molded bioplastics and a recycled polypropylene blend. Sustainability.

[12] Yoon S.H., Padmanabha P., Cha N.G., Mead J.L., Barry C.M.F. (2011). Evaluation of vacuum venting for micro-injection molding. Int. Polym. Process..

[13] Sorgato M., Masato D., Lucchetta G. (2017). Effect of vacuum venting and mold wettability on the replication of micro-structured surfaces. Microsyst. Technol..

[14] O’Toole L., Kang C.-W., Fang F.-Z. (2021). Precision micro-milling process: State of the art. Adv. Manuf..

[15] Chen Y., Hu S., Li A., Cao Y., Zhao Y., Ming W. (2023). Parameters Optimization of Electrical Discharge Machining Process Using Swarm Intelligence: A Review. Metals.

[16] https://www.mdpi.com/journal/micromachines/special_issues/LS77E2HJ2C.

